# Interspecies Jumping of Bat Coronaviruses

**DOI:** 10.3390/v13112188

**Published:** 2021-10-29

**Authors:** Antonio C. P. Wong, Susanna K. P. Lau, Patrick C. Y. Woo

**Affiliations:** Department of Microbiology, Li Ka Shing Faculty of Medicine, The University of Hong Kong, Pokfulam, Hong Kong, China; antonwcp@connect.hku.hk

**Keywords:** interspecies jumping, bat, coronavirus, outbreak, epidemic, pandemic, SARS, MERS, SADS, COVID-19

## Abstract

In the last two decades, several coronavirus (CoV) interspecies jumping events have occurred between bats and other animals/humans, leading to major epidemics/pandemics and high fatalities. The SARS epidemic in 2002/2003 had a ~10% fatality. The discovery of SARS-related CoVs in horseshoe bats and civets and genomic studies have confirmed bat-to-civet-to-human transmission. The MERS epidemic that emerged in 2012 had a ~35% mortality, with dromedaries as the reservoir. Although CoVs with the same genome organization (e.g., *Tylonycteris* BatCoV HKU4 and *Pipistrellus* BatCoV HKU5) were also detected in bats, there is still a phylogenetic gap between these bat CoVs and MERS-CoV. In 2016, 10 years after the discovery of *Rhinolophus* BatCoV HKU2 in Chinese horseshoe bats, fatal swine disease outbreaks caused by this virus were reported in southern China. In late 2019, an outbreak of pneumonia emerged in Wuhan, China, and rapidly spread globally, leading to >4,000,000 fatalities so far. Although the genome of SARS-CoV-2 is highly similar to that of SARS-CoV, patient zero and the original source of the pandemic are still unknown. To protect humans from future public health threats, measures should be taken to monitor and reduce the chance of interspecies jumping events, either occurring naturally or through recombineering experiments.

## 1. Introduction

Coronaviruses (CoVs) are positive-sense, single-stranded RNA viruses that infect mammals and birds. CoVs are classified into four genera, *Alphacoronavirus* (previously group 1 CoV), *Betacoronavirus* (previously group 2 CoV), *Gammacoronavirus* (previously group 3 CoV) and *Deltacoronavirus*, of which *Betacoronavirus* is subclassifed into five subgenera, namely *Embecovirus* (the traditional betaCoVs), *Sarbecovirus*, *Merbecovirus*, *Nobecovirus* and *Hibecovirus*. From our present knowledge, bats are the hosts of only *Alphacoronavirus* and the subgenera *Sarbecovirus*, *Merbecovirus* and *Nobecovirus* of *Betacoronavirus* [1]. The size of a CoV varies from 26 kb to 31 kb [2]. Around two-third of the genome is occupied by open reading frame 1ab (ORF1ab), which encodes 15–16 non-structural proteins (nsps), including papain-like protease, chymotrypsin-like protease, RNA-dependent RNA polymerase (RdRp), helicase, etc. [3]. The remaining one-third of the genome encodes the spike (S), envelope (E), membrane (M) and nucleocapsid (N) proteins [3,4]. Furthermore, a variable number of additional accessory genes that code for accessory proteins may also be present downstream to ORF1ab. These accessory proteins do not display significant homologies to those from other more distantly related CoV species [5]. Mutations, insertions and deletions are common in these accessory genes [6,7,8,9]. Although the accessory proteins are generally considered non-essential for viral replication, most of their functions are not fully understood [10,11,12,13,14,15,16].

The first step of interspecies jumping requires binding of a CoV to the cells of a new host [17]. The virus is then internalized and if it can utilize the machinery of the new host for replication, it may be able to perpetuate in the host [18]. Further adaptation usually requires additional mutations in the viral genome [19]. The S protein is located on the surface of the virus. During an infection, it binds to the receptor of the host, resulting in virus entry [20]. Therefore, it is undeniably the most crucial protein that determines whether a particular CoV can infect a specific host, and mutations in the *S* gene, either naturally or through recombineering experiments, may lead to potential interspecies jumping [21,22,23,24,25,26,27]. The S protein consists of two parts, S1 and S2. S1 is the longer (but more variable in length e.g., ~685 amino acids in SARS-CoV and ~750 amino acids in MERS-CoV) and exposed part that contains the receptor binding domain (RBD), whereas S2 (~600 amino acids) is the shorter and more conserved transmembrane portion [28,29,30]. In some CoVs, the S protein is cleaved at the S1/S2 junction and modification of the amino acids at the cleavage site would affect the infectivity of the virus [31]. In addition to the S protein, other accessory proteins may also play important roles in subsequent adaptation to the new host after interspecies jumping [9,11,32,33,34].

In this article, we review the probable CoV interspecies jumping events that are known to have occurred between bats and other animals/humans, most of which have resulted in major epidemics and pandemics and frighteningly high fatalities. Transmission and adaptation of CoVs between different bat species, such as the jumping of Bat coronavirus HKU10 between Leschenault’s rousettes (*Rousettus leschenaulti*) and Pomona leaf-nosed bats (*Hipposideros Pomona*), will not be included [35].

## 2. SARS-CoV: The Classical Example of Bat-To-Animal-To-Human Interspecies Jumping

The SARS epidemic in 2002/2003 had a fatality of around 10% among the ~8000 laboratory confirmed cases [36]. Patient zero of the epidemic was believed to be a cook who had handled animals in a restaurant in southern China that served meat from wildlife [37]. After circulating in mainland China for a few months, it was transmitted to Hong Kong in early 2003 by a Chinese professor who stayed in a hotel in Hong Kong, a busy international financial hub and the gateway of China [38,39,40]. During the same visit, the Chinese professor has also infected other residents in the hotel, who efficiently spread the infection from Hong Kong to different parts of the world when they went back to their corresponding home countries [39,41].

**Figure 1:**
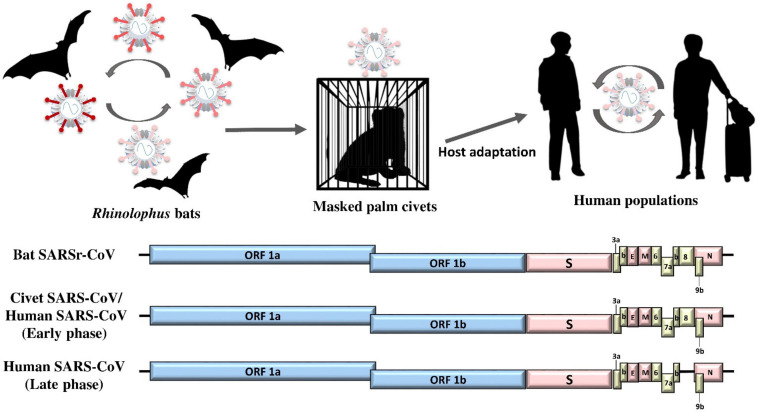
Overview of interspecies transmission events of SARS epidemic in 2003 and genome organizations of SARSr-CoV from different hosts. Arrows indicate the direction of viral interspecies jumping and viral molecular evolution between different host species. Circular arrows indicate the circulation of virus within the population. ORF1a and ORF1b are represented by blue boxes, structural proteins by pink boxes, and accessory proteins by yellow boxes.

Shortly after the isolation of SARS-CoV, the virus was discovered in palm civets in wet markets in the Guangdong Province [8]. However, multiple lines of evidence showed that the civets were probably just the intermediate hosts, but not the ultimate ancestor of SARS-CoV (Figure 1) [42,43,44]. One part of the evidence was that only civets in the farms, but not those in the wild, were found to harbor the virus, indicating that the civets had only acquired the virus recently, instead of being its reservoir for a long time (Figure 1) [42]. Intensive surveillance research in Hong Kong was therefore carried out and revealed that SARSr-CoVs were present in the fecal samples of Chinese horseshoe bats (*Rhinolophus sinicus*) that resided in caves and water tunnels in Hong Kong [45]. Other studies in China have also found these SARSr-CoVs in different horseshoe bat species, including greater horseshoe bats (*Rhinolophus ferrumequinum*), big-eared horseshoe bats (*Rhinolophus macrotis*), least horseshoe bats (*Rhinolophus pusillus*), intermediate horseshoe bats (*Rhinolophus affinis*), and Blasius’s horseshoe bats (*Rhinolophus blasii*), as well as Stoliczka’s Asian trident bats (*Aselliscus stoliczkanus*) and wrinkled-lipped free-tailed bats (*Chaerephon plicata*), mainly in various provinces in southern China but occasionally in other countries [17,33,46,47,48,49]. Interestingly, for reasons not fully understood, there have been no more human SARS cases since July 2003, except for a number of laboratory acquired cases in China in late 2003 to early 2004 [50].

**Figure 2:**
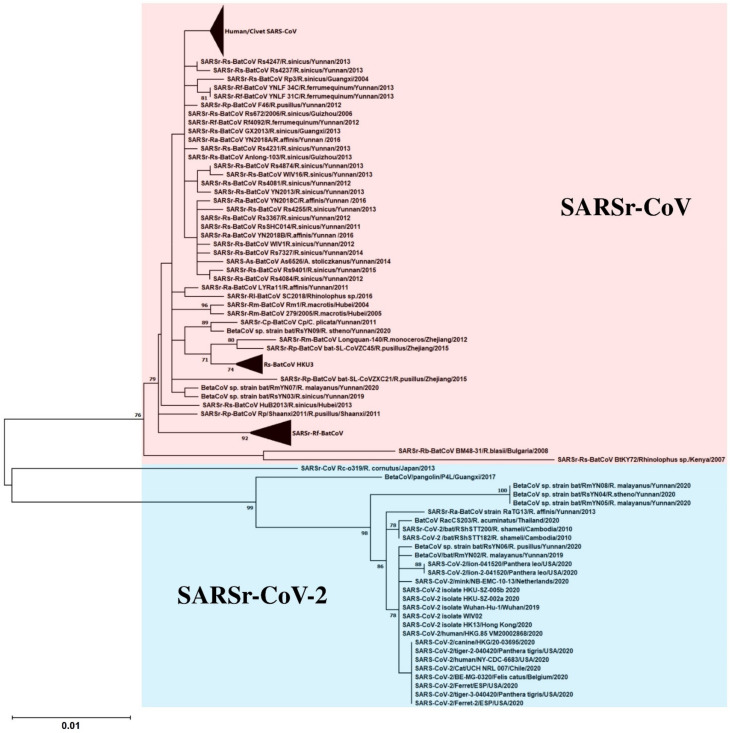
Maximum-likelihood phylogeny based on the nsp12 amino acid sequences of selected SARSr-CoVs. A Jones–Taylor–Thornton (JTT) model of amino acid substitution was used in the analysis with a discrete gamma (γ) distribution and the assumption that a certain fraction of sites is evolutionarily invariable (+I). 1000 trees were set for bootstrap values calculation. All bootstrap values and the scale bar indicating the number of amino acid substitutions per site are shown. Viral strains highlighted in red color represents the SARSr-CoV cluster; blue color represents the SARSr-CoV-2 cluster.

SARSr-CoVs from humans, civets and bats belong to the subgenus *Sarbecovirus* of *Betacoronavirus*. The size of their genomes is around 29,750 bp. In addition to the basic genome backbone 5′-ORF1ab-S-E-M-N-3′, several ORFs that encode accessory proteins, including ORF3a, ORF3b, ORF6, ORF7a, ORF7b, ORF8 and ORF9b, are present (Figure 1 and Table 1) [51]. Among the proteins, the S protein is the most crucial in dictating the interspecies jumping. The receptor for SARS-CoV in humans is angiotensin-converting enzyme 2 (ACE2) [29,52,53]. Not unexpectedly, the S proteins from most SARSr-CoV strains found in the horseshoe bats were not able to bind the ACE2 in humans [45,54]. However, it was shown recently that the S proteins of some SARSr-CoV strains found in bats from the Yunnan province in China were able to bind the ACE2 in humans, suggesting that this was an important step in the evolution of the virus towards jumping to humans [24,25,26]. Since the S protein is so crucial in determining infection across difference animal species, two groups have performed recombineering experiments involving the S protein to ascertain its importance. Since the SARS epidemic, a large diversity of bat SARSr-CoVs has been found in various species of horseshoe bats, with complete genome sequences available, facilitating such experiments (Figure 2). One of the bat SARSr-CoV strains, named WIV1, successfully isolated in Vero E6 cells by a group of Chinese scientists in Wuhan in 2013, was shown to be able to utilize the ACE2 receptors of humans, civets and Chinese horseshoe bats for host cell entry, implying its potential to have broad species tropism [26]. In 2015, another group, in the USA, extended the study of potential interspecies transmission by developing a reverse genetics system for generating chimeric SARSr-CoV [25]. They cloned the S gene obtained from bat SARSr-CoV strain SHC014, predicting that its S protein could bind ACE2 as do those SARSr-CoV strains that possessed similar S protein sequences with the critical amino acid residues for ACE2-binding, to a mouse-adapted SARSr-CoV strain MA15 backbone. The recombinant SARSr-CoV strain SHC014-MA15 was able to multiply in mouse DBT cell lines expressing human, civet or bat ACE2, indicating that the S protein of bat SARSr-CoV strain SHC014 was able to utilize different orthologs of human ACE2 as receptors. Furthermore, the recombinant SARSr-CoV strain SHC014-MA15, but not the original SARSr-CoV strain SHC014, was also able to infect BALB/c mice with substantial weight loss observed, suggesting the potential of interspecies transmission of the recombinant virus in laboratory settings. After this initial success, the research group further generated another chimeric SARSr-CoV by cloning the *S* gene of SARSr-CoV strain WIV1 into the mouse-adapted SARSr-CoV strain MA15 backbone and rescued in Vero cells. Animal experiments suggested that both the SARSr-CoV strain WIV1 and the recombinant SARSr-CoV strain WIV1-MA15 could not significantly infect BALB/c mice [24]. However, they showed subsequently that the SARSr-CoV strain WIV1 was able to cause attenuated infection in transgenic mice expressing human ACE2, indicating that the species barrier could be overcome by altering the target host cellular expression profile genetically.

## 3. MERS-CoV: Is It from Bat Again?

Bats were not known to be hosts for CoVs before SARS. After the SARS epidemic and the discovery that bats are the ancestor of SARSr-CoVs, at least 17 novel CoVs have been found in various species of bats [17,62,63,64]. In additional to the discovery of these previously unknown bat CoVs, between the SARS outbreak in 2002/2003 and the emergence of MERS in 2012, there have been many other advances in our knowledge and understanding in CoV diversity and phylogeny [62,63,64]. A fourth genus of CoV, *Deltacoronavirus*, has been discovered, in addition to the three genera, *Alphacoronavirus*, *Betacoronavirus* and *Gammacoronavirus* that have been known for decades [65,66,67,68]. *Betacoronavirus* was also subclassified into the four subgenera *Embecovirus* (the traditional betaCoVs), *Sarbecovirus*, *Merbecovirus* and *Nobecovirus* during this period [64].

The MERS epidemic that emerged in 2012 had a frightening mortality of around 35% among the laboratory confirmed cases. Patient zero was a 60-year-old Saudi Arabian male from Jeddah in Saudi Arabia, from whom the first MERS-CoV strain, that belonged to the subgenus *Merbecovirus* in *Betacoronavirus*, was isolated [69,70]. Although the total number of laboratory-confirmed cases so far is only around 2500, the epidemic is still ongoing. Patients who developed MERS were either from the Middle East or had recently travelled there or were in contact with returning travelers from the Middle East. So far, the largest cluster of cases was in Saudi Arabia, involving over 600 patients; whereas outside the Middle East, the largest cluster occurred in South Korea, which originated from a patient who visited the Middle East and brought the infection back to his home country, infecting a total of 184 patients [71,72,73].

**Figure 3:**
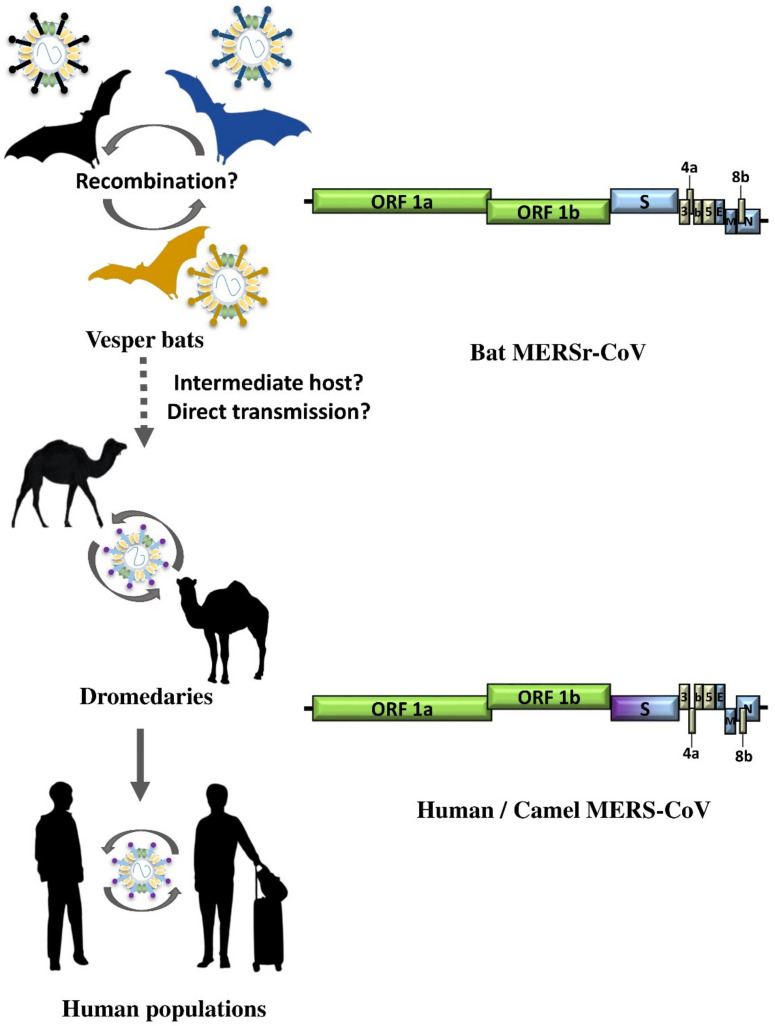
Overview of interspecies transmission events of MERS epidemic since 2012. Arrows indicate the direction of viral interspecies jumping between different host species. Circular arrows indicate the circulation of virus within the population. Dot arrows indicate the viral interspecies jumping event remains to be elucidated. ORF1a and ORF1b are represented by green boxes, structural proteins by blue and purple boxes, and accessory proteins by yellow boxes.

Shortly after the emergence of the MERS epidemic in humans, intensive surveillance studies have found that MERS-CoV was present in the respiratory samples of dromedaries in the Middle East and North Africa, suggesting that it was the reservoir of the virus (Figure 3) [74,75,76]. Infection was acquired through direct or indirect contact with the camels [77]. Further studies also showed that the virus mainly infected dromedary calves and the sero-positivity rate increased with the age of the dromedaries [78]. Although MERS-CoV is not present in wild Bactrian camels, those that were intranasally inoculated with the virus did develop symptoms and the virus could also be isolated from their respiratory samples as well as being seroconverted [79]. Moreover, we have also detected MERS-CoV neutralizing antibodies in Bactrian camels in Dubai, The United Arab Emirates. These indicated that Bactrian camels could also be potential reservoirs of MERS-CoV [80].

**Figure 4:**
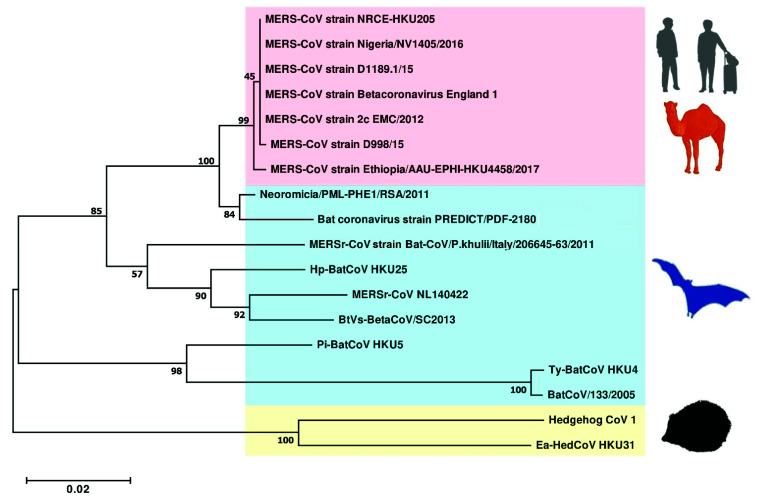
Maximum-likelihood phylogeny based on the nsp12 amino acid sequences of selected MERSr-CoVs and merbecoviruses. A Jones–Taylor–Thornton (JTT) model of amino acid substitution was used in the analysis with a discrete gamma (γ) distribution. 1000 trees were set for bootstrap values calculation. All bootstrap values and the scale bar indicating the number of amino acid substitutions per site are shown. Viral strains highlighted in red color represents the MERSr-CoV cluster, blue color represents the bat merbecoviruses cluster, yellow color represents the hedgehog merbecoviruses cluster.

The size of the MERS-CoV genome is around 30,110 bp. When it was discovered, it was found to be most closely related to *Tylonycteris* bat CoV HKU4 (*Ty*-BatCoV HKU4), *Pipistrellus* bat CoV HKU5 (*Pi*-BatCoV HKU5), that we discovered from the fecal samples of lesser bamboo bat (*Tylonycteris pachypus*) and Japanese pipistrelle (*Pipistrellus abramus*), respectively, in 2006 [64,81,82]. MERS-CoV, *Ty*-BatCoV HKU4, and *Pi*-BatCoV HKU5 all belong to the subgenus *Merbecovirus* in *Betacoronavirus*, and they share similar genome size and organization (Figure 3) [81,82]. In addition to the basic genome backbone 5′-ORF1ab-S-E-M-N-3′, several ORFs that encode accessory proteins, including ORF3, ORF4a, ORF4b, ORF5 and ORF8b, are present (Figure 3 and Table 2) [70]. Despite these similarities, MERS-CoV, *Ty*-BatCoV HKU4 and *Pi*-BatCoV HKU5 are not as closely related to each other as bat, civet and human SARSr-CoVs [81,82]. Therefore, it has boosted interest in the search for additional merbecoviruses, with the aim of identifying ancestors that are more closely related to MERS-CoV than *Ty*-BatCoV HKU4 and *Pi*-BatCoV HKU5. In the past few years, several merbecoviruses were discovered in other bat species, as well as hedgehogs. These include *Hypsugo pulveratus* bat coronavirus HKU25, Neoromicia/PML-PHE1/RSA/2011, BatCoV PREDICT/PDF-2180, MERSr-CoV isolates NL140422, Hedgehog coronavirus 1, *Erinaceus amurensis* hedgehog coronavirus HKU31, BtVs-BetaCoV SC2013 and MERSr-CoV isolate Bat-CoV/P.khulii/Italy/206645-63/2011 (Figure 4) [27,83,84,85,86,87,88,89].

The receptor of MERS-CoV is human dipeptidyl peptidase (hDPP4) [30,96]. Detailed phylogenetic analysis and comparison of the S proteins of MERS-CoV, *Ty*-BatCoV HKU4 and *Pi*-BatCoV HKU5 revealed that the S protein of MERS-CoV is more related to that of *Ty*-BatCoV HKU4 and *Pi*-BatCoV HKU5 [81,82]. It was also shown that the S protein of *Ty*-BatCoV HKU4, but not that of *Pi*-BatCoV HKU5, can bind hDPP4 [97,98]. Recently, we have isolated *Ty*-BatCoV HKU4 using Caco-2 and Huh-7 cells and found that it was able to infect transgenic mice expressing hDDP4, showing pathologies similar to, but less severe than, those infected with MERS-CoV [99]. These indicated that *Ty*-BatCoV HKU4 or its close relative is likely sharing the common ancestor of MERS-CoV.

## 4. *Rhinolophus* Bat CoV HKU2: Another CoV from Horseshoe Bats

**Figure 5:**
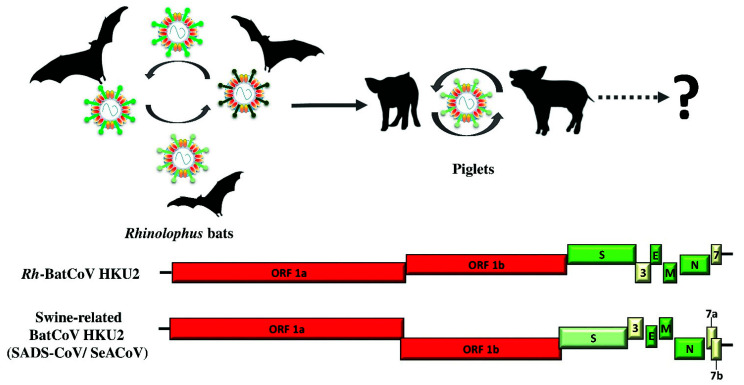
Overview of interspecies transmission events of swine-related BatCoV HKU2 (SADS) outbreak since 2017. Arrows indicate the direction of viral interspecies jumping between different host species. Circular arrows indicate the circulation of virus within the population. Dot arrows indicate the viral interspecies jumping event remains to be elucidated.

In 2006, in the same study in which we described the discovery of *Ty*-BatCoV HKU4 and *Pi*-BatCoV HKU5, we also first reported the discovery of *Rhinolophus* bat CoV HKU2 (*Rh*-BatCoV HKU2) from 8.3% of the fecal samples of Chinese horseshoe bats (*R. sinicus*) in Hong Kong, sharing the same host as bat SARSr-CoV (Figure 5) [100]. Unlike the SARSr-CoVs and MERSr-CoVs that are betaCoVs, *Rh*-BatCoV HKU2 belongs to another genus, *Alphacoronavirus*, which is highly diversified [5,62,63,101,102,103,104]. After *Rh*-BatCoV HKU2 was discovered in Chinese horseshoe bats from Hong Kong, similar to the situation of SARSr-CoVs, it was also found in intermediate horseshoe bats, Chinese horseshoe bats, least horseshoe bats, king horseshoe bats, big-eared horseshoe bats, greater horseshoe bats, eastern bent-wing bats, great roundleaf bats, and Pratt’s roundleaf bats from mainland China, and Shamel’s horseshoe bats from Thailand, respectively [54,105,106,107]. However, the genomes of these strains, except one from an intermediate horseshoe bat (isolate 160,660) and one from a greater horseshoe bat (isolate BtRf-AlphaCoV/YN2012) in China, were not sequenced and analyzed [105,107].

The size of the *Rh*-BatCoV HKU2 genome is only around 27 kb, the smallest among all the known CoVs [100]. In addition to the standard genome organization of 5′-ORF1ab-S-E-M-N-3′, two accessory genes, ORF3 and ORF7 of unknown function to date, were observed (Figure 5) [100]. Notably, the S protein of *Rh*-BatCoV HKU2 has only 1128 amino acid residues, the shortest among all known CoVs. Phylogenetic analysis also showed that this *Rh*-BatCoV HKU2 S protein forms a distinct branch, suggesting that it may have a unique evolutionary pathway [100]. It is of interest that in 2015 and 2017, two novel alphacoronaviruses, Lucheng Rattus norvegicus rat coronavirus (LRNV) and Wénchéng shrew virus (WESV) were reported in brown rat (*Rattus norvegicus*) and Asian house shrews (*Suncus murinus*) from mainland China [108,109]. Intriguingly, these two novel alphacoronaviruses also have short S proteins, although distinct from that of *Rh*-BatCoV HKU2 (only around 38–42% amino acid identity) as well as those of other known alphacoronaviruses [108,109].

In 2016, 10 years after the first discovery of *Rh*-BatCoV HKU2 in Chinese horseshoe bats, a series of fatal swine disease outbreaks affecting newborn piglets was reported in several pig farms in Qingyuan of the Guangdong province in Southern China [107,110,111,112]. Porcine epidemic diarrhea virus (another alphaCoV) was initially considered as the causative agent for these outbreaks but subsequently it was no longer detected in the dead pigs despite the persistence of the outbreaks and increasing fatality [107]. The possibilities of other swine-related viruses, such as parvovirus, pseudorabies virus, rotavirus, foot and mouth disease virus, classical swine fever virus, sapelovirus, swine influenza virus, etc. were also excluded. Finally, by performing next generation sequencing on the piglet intestinal samples, *Rh*-BatCoV HKU2 was unexpectedly identified, and its causative role was subsequently confirmed by viral isolation in tissue culture and other experiments to fulfil Koch’s postulates (Figure 5) [107,110]. Two groups of Chinese scientists named this *Rh*-BatCoV HKU2 found in the piglets as swine enteric alphacoronavirus (SeACoV) and swine acute diarrhea syndrome coronavirus (SADS-CoV) respectively, pending validation by the ICTV [107,111,113]. After the initial outbreaks, another strain was detected in seven pig farms in the Fujian Province in 2018, and re-emergence of the fatal swine disease due to this virus was also reported in February 2019, leading to around 2000 piglet deaths in a pig farm of the Guangdong Province, near the origin of the previous outbreak [114]. So far, this novel swine enteric disease has not been reported outside China. However, the sporadic outbreaks of the disease suggest that multiple interspecies jumping events might have occurred between the bat and swine populations, similar to the case of MERS-CoV with polyphyletic origin [113,115].

**Figure 6:**
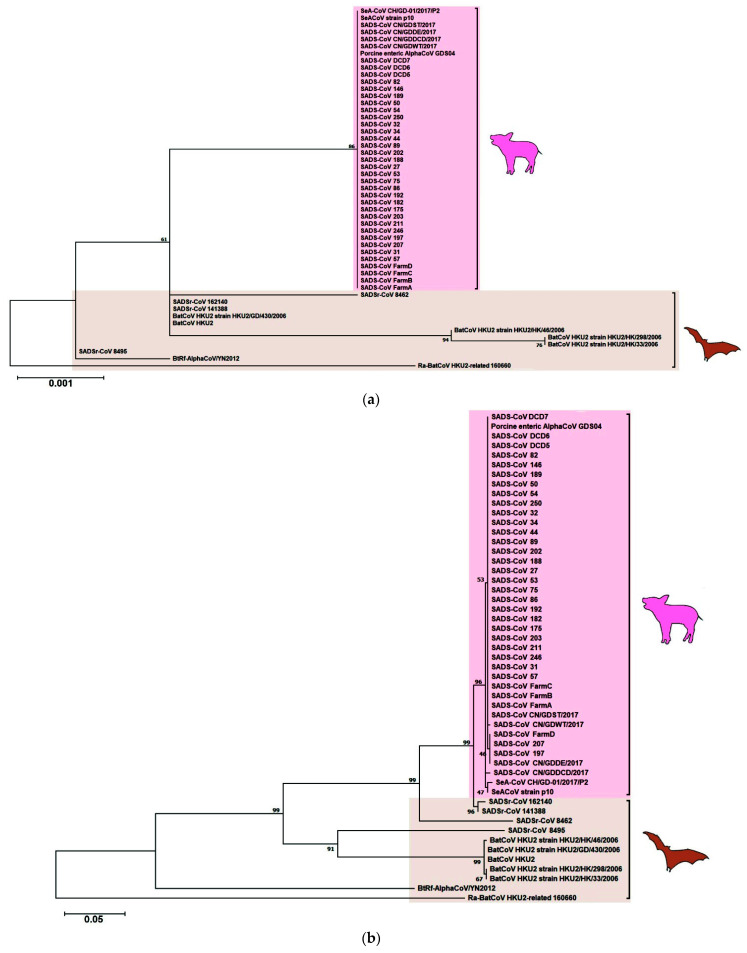
Maximum-likelihood phylogeny based on the amino acid sequences of selected BatCoV HKU2 and swine-related BatCoV HKU2 (**a**) nsp12 and (**b**) S1 domains. (**a**) a Jones–Taylor–Thornton (JTT) model of amino acid substitution and (**b**) a Whelan–Goldman matrix (WAG) model with a discrete gamma (γ) distribution of amino acid substitution was used in the respective analyses. 1000 trees were set for bootstrap values calculation. All bootstrap values and the scale bars indicating the number of amino acid substitutions per site are shown. Viral strains highlighted in pink color represents Table 2 cluster; brown color represents the BatCoV HKU2 cluster.

Being the same species, there is an overall 87–95% nucleotide sequence identity between SeACoV/SADS-CoV and *Rh*-BatCoV HKU2 [107,110,111]. However, the S gene of SeACoV/SADS-CoV shows only around 73–86% amino acid sequence identity to that of *Rh*-BatCoV HKU2, indicating that significant changes in the S protein are required for its binding to the corresponding receptor in pigs, leading to interspecies jumping. This theory was further supported by the discovery of four additional *Rh*-BatCoV HKU2 strains, named SADSr-CoV strains 141,388, 162,140, 8495 and 8462, collected from intermediate horseshoe bats and unspecified horseshoe bats in 2013–2016 from the Guangdong province of China (Figure 6) [107]. These four strains shared 96–98% overall nucleotide sequence identity with SeACoV/SADS-CoV. Moreover, the S genes of SADSr-CoV strains 141,388 and 162,140 shared > 98% amino acid sequence identity with that of SeACoV/SADS-CoV [107]. Importantly, an additional accessory gene, ORF7b, was found to be present only in SeACoV/SADS-CoV and some of the recently discovered *Rh*-BatCoV HKU2 strains (SADSr-CoV strains 162,140 and 8462), suggesting that multiple steps might have occurred for the interspecies jumping event between bats and pigs to happen (Figure 5) [107].

## 5. SARS-CoV-2: The SARS-CoV Story Retold?

**Figure 7:**
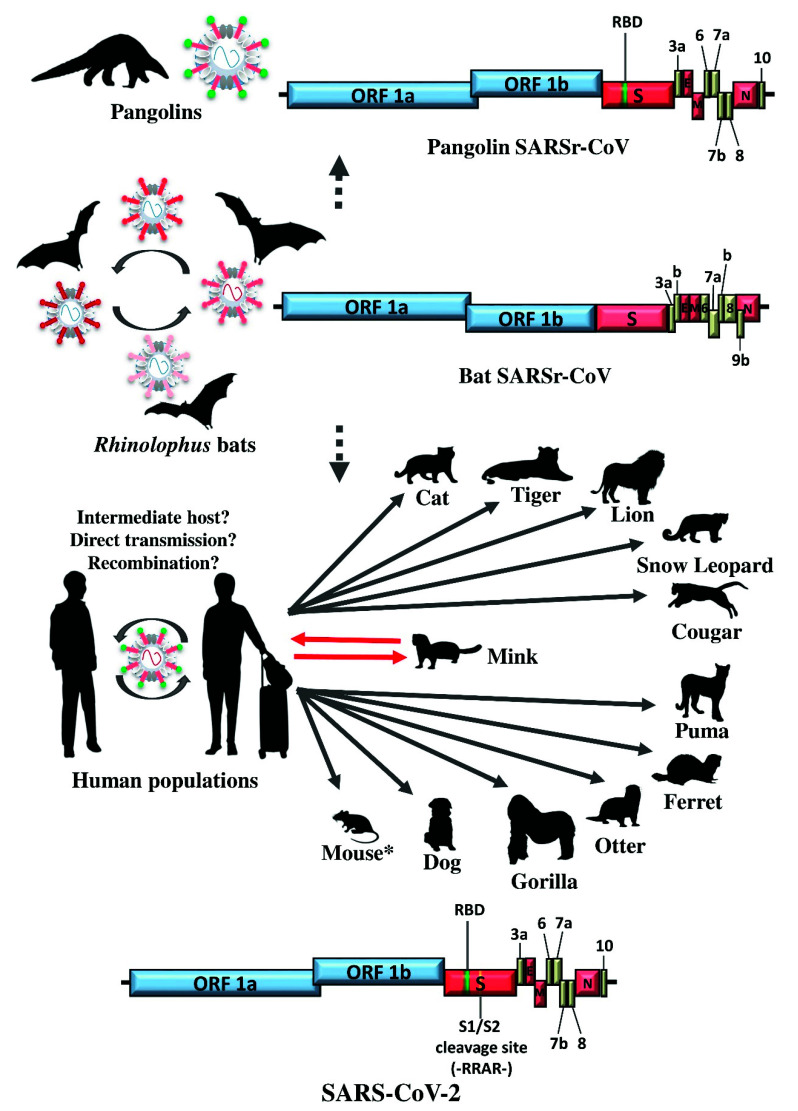
Overview of interspecies transmission events of COVID-19 pandemic since 2019. Arrows indicate the direction of viral interspecies jumping and viral molecular evolution between different host species. Circular arrows indicate the circulation of virus within the population. Dot arrows indicate the viral interspecies jumping event remains to be elucidated. Red arrows indicate the bi-directionality of interspecies jumping events. Asterisk labeled animal represents the laboratory interspecies jumping event through host adaption. ORF1a and ORF1b are represented by blue boxes, structural proteins by red boxes, and accessory proteins by yellow boxes. The RBD and the -RRAR- cleavage site are highlighted in green and yellow respectively.

In late 2019, an outbreak of pneumonia cases occurred in Wuhan, China. The infection rapidly spread to every single country in the world in 2020, leading to countless people losing their lives or having permanent lung damage [116]. By 1 October 2021, there have already been more than 5 million laboratory confirmed fatalities; and the infection, named COVID-19 by the World Health Organization, is still raging worldwide, despite the availability of reasonably effective mRNA vaccines developed by Moderna, Inc and Biopharmaceutical New Technologies (BioNTech SE), as well as recombinant adenoviral vector encoding SARS-CoV-2 spike protein vaccines developed by AstraZeneca plc and Johnson & Johnson (J&J) within one year of the beginning of the pandemic [117,118,119]. However, patient zero is still unknown and neither is the original source of the pandemic [120,121,122]. A CoV closely related to the SARSr-CoVs, belonging to the subgenus *Sarbecovirus* of *Betacoronavirus*, has been isolated from COVID-19 patients. The CoV was subsequently named as SARS-CoV-2, distinguishing it from the SARS-CoV that caused the SARS epidemic in 2003 [123]. In contrast to SARS-CoV, SARS-CoV-2 has been circulating in humans for almost two years already. Several factors have contributed to the enormous differences in the scale and consequence of the SARS and COVID-19 outbreaks. First, the emergence of SARS-CoV-2 from an unknown source posed difficulties in conducting blocking tactics. There is no concrete scientific evidence so far showing whether the interspecies transmission was directly from bats to humans or if any intermediate animal host was involved [124]. Second, many countries have missed the chance of implementing early infection control tactics, such as wearing masks, quarantine and travel restrictions, leading to uncontrollable viral transmission links established globally instead of restricting the early outbreak within a target population [125]. Third, SARS-CoV-2 has displayed highly human-adapted characteristics and a rapid transmission rate within the human population since its first emergence [126,127]. Epidemiologically, the 7.8 billion human population, which exceeds the critical community size, has provided a large and stable maintenance population for SARS-CoV-2 to circulate and adapt. The emergence of many variants of interest and concern through mutation has made it difficult to achieve complete eradication through reservoir control measures, such as global mass vaccination, as it has been shown that some of the novel variants may escape existing available vaccine protection [128,129,130,131]. Therefore, it is possible that humans may become a new permanent component contributing to the reservoir of SARS-CoV-2, which would be the fifth persisting human CoV (HCoV) after HCoV-OC43, HCoV-229E, HCoV-NL63 and HCoV-HKU1 [132,133,134,135,136,137]. Although no animal reservoir was identified and no animal-to-human transmission documented, the virus has been shown to infect a number of mammals, including dogs, domestic cats, lions, tigers, snow leopards, cougars, pumas, ferrets, minks and gorillas globally (Figure 2 and Figure 7) [138,139,140,141]. However, it appears that most of these animal infections, except for minks which can transmit SARS-CoV-2 back to human, were dead-end infections, as they have not yet resulted in any further spread of the disease, suggesting that these animals belong to the non-maintenance population (Figure 7).

The genome size of SARS-CoV-2 is around 29,903 kb, similar to that of other SARSr-CoVs [116,120]. It also shares similar genomic organization as the SARSr-CoVs found in various species of horseshoe bats, consisting of the basic genome backbone 5′-ORF1ab-S-E-M-N-3′ and a similar set of ORFs that encode accessory proteins, including ORF3a, ORF6, ORF7a, ORF7b, ORF8 and ORF10 (Figure 7) [120]. Despite these similarities, the difference in the sequences of the individual genes, particularly the S protein, have led to the differential phenotypes and virulence between SARS-CoV-2 and the other SARSr-CoVs found in bats, civets and human in the previous epidemic, and hence different clinical manifestations of SARS and COVID-19 [142,143,144,145,146,147]. In fact, the RBD region of the S protein in SARS-CoV-2 only shares around 74–77% amino acid identity with those of human, civet and bat SARSr-CoVs that were previously reported before the COVID-19 outbreak, although these SARSr-CoVs can all utilize human ACE2 despite their different binding affinities [143,148,149,150,151,152]. Shortly after the beginning of the pandemic, two independent groups have found in their surveillance studies that SARSr-CoVs were detected in pangolins in the Guangdong province of southern China and that the RBD region of the S protein in SARS-CoV-2 shares the closest relationship (~97% amino acid identity) with those observed in these pangolin SARSr-CoVs [153,154]. In addition, a recent pre-print research article has described the discovery of a bat SARSr-CoV strain BANAL-20-52 from the Indochinese peninsula, which also carries an RBD region with ~97% amino acid identity to that of SARS-CoV-2 [155].

Until now, undeniably one of the most intriguing aspects in the genome of SARS-CoV-2 is located at the S1/S2 junction. A unique, short amino acid (PRRA) insertion is found within the S gene of SARS-CoV-2, leading to a potential furin cleavage site (RRAR) between the S1 and S2 subunits (Figure 7) [31,152]. Such an insertion allows the SASRS-CoV-2 S protein to undergo proteolytic cleavage, resulting in cell-cell fusion enhancement and contributes to pathogenesis [31,156,157,158]. It is of note that the additional cleavage site in the S gene has not been observed in any bat SARSr-CoV strains discovered so far since 2003, including those recently discovered in southeast Asia [155,159,160,161,162,163], nor in any pangolin SARS-CoV-2 strains [153,154,161]. This has raised concerns in both the scientific community and the general public globally, pending further research into its possible origin.

## 6. Concluding Remarks

Due to the absence of existing immunity, infections caused by a novel virus as a result of recombination, reassortment and/or mutations and subsequent interspecies jumping, have often led to epidemics that can be highly lethal. In CoVs, this has been well exemplified in the last two decades in three independent pandemics, two originated in China and one from the Middle East. The first two, the SARS and MERS epidemics in 2003 and 2012, respectively, have been confirmed to be transmitted from animal reservoirs to humans [164,165,166]. Thorough and scientific investigations on the origin of COVID-19 and the potential threat of *Rh*-BatCoV HKU2-related swine disease are yet to be completed [167,168]. Recent research advancement in viral pathogenesis studies by artificial manipulation of viral genomes like recombineering experiments may lead to the generation of novel pathogens with unpredictable virulence [23]. Therefore, performing animal surveillance studies, controlling animal food markets, implementing good hygienic measures during animal contacts, etc. are essential for mitigating possible future outbreaks while having a global concerted effort to regulate and monitor viral genetic engineering experiments is of crucial importance to secure a safe research environment [21].

## Figures and Tables

**Table 1:** Putative functions of SARS-CoV accessory proteins.

Accessory Protein	Putative Function(s)
ORF3a	Induces chemokines production, including RANTES and IL-8; activates NF-κB and JNK [55]
ORF3b	Induces necrosis or apoptosis; upregulates cytokines through RUNX1 [56]
ORF6	Formation of double membrane vesicles [57]; inhibits nuclear import of STAT1 (IFN-β antagonist) [58]
ORF7a	Activates NF-κB and JNK [55]; induces apoptosis; inhibits host protein translation; activates p38 MAPK [59]
ORF7b	Remain to be elucidated
ORF8	Activates ATF6; upregulates ER-resident chaperons [60]
ORF9b	Suppresses IFN responses by indirectly degrading MAVS [61]

RANTES: regulated on activation, normal T cell expressed and secreted; IL-8: interleukin-8; NF-κB: nuclear factor kappa-light-chain-enhancer of activated B cells; JNK: c-Jun N-terminal kinases; RUNX1: runt-related transcription factor 1; STAT1: signal transducer and activator of transcription 1; MAPK: mitogen-activated protein kinase; ATF6: activate activating transcription factor 6; ER: endoplasmic reticulum; IFN: interferon; MAVS: mitochondrial antiviral-signaling protein.

**Table 2:** Putative functions of MERS-CoV accessory proteins.

Accessory Protein	Putative Function(s)
ORF3	Inhibits type I IFN responses [90]
ORF4a	Inhibits MDA5-mediated IFN activation [91]; interferes in PKR-mediated antiviral stress responses [92]
ORF4b	Inhibits type I IFN responses by interacting with TBK1 and IKKε [93]
ORF5	Reduces NF-κB activation and inflammatory cytokines [90]
ORF8b	Inhibits IFN-β promoter activity by RIG-I [94]; suppresses IKKε activated type I IFN responses [95]

IFN: interferon; MDA5: melanoma differentiation-associated protein 5; PKR: protein kinase R; TBK1: TANK binding kinase 1; IKKε: IκB kinase epsilon; NF-κB: nuclear factor kappa-light-chain-enhancer of activated B cells; RIG-I: retinoic acid-inducible gene I.

## Data Availability

Data available in publicly accessible repositories. The data presented in this study are openly available in National Center for Biotechnology Information and GISAID Initiative.

## Abbreviations

ACE2	Angiotensin converting enzyme 2
AlphaCoV	Alphacoronavirus
ATF6	Activate activating transcription factor 6
BetaCoV	Betacoronavirus
BioNTech	Biopharmaceutical New Technologies
bp CoV	Base pair Coronavirus
ER	Endoplasmic reticulum
E	Envelope
γ	Gamma
HCoV	Human coronavirus
hDPP4	Human dipeptidyl peptidase 4
I	Invariable
IKKε	IκB kinase epsilon
IFN	Interferon
IL	Interleukin
J&J	Johnson & Johnson
JNK	c-Jun N-terminal kinases
JTT	Jones-Taylor-Thornton
kb	kilobase
MAPK	Mitogen-activated protein kinase
MAVS	Mitochondrial antiviral-signaling protein
MDA5	Melanoma differentiation-associated protein 5
M	Membrane
MERS-CoV	Middle East respiratory syndrome coronavirus
NF-κB	Nuclear factor kappa-light-chain-enhancer of activated B cells
N	Nucleocapsid
ORF	Open reading frame
PCR	Polymerase chain reaction
PKR	Protein kinase R
Pi	Pipistrellus
RANTES	Regulated on activation, normal T cell expressed and secreted
RBD	Receptor binding domain
RdRp	RNA-dependent RNA polymerase
Rh	Rhinolophus
RIG-I	Retinoic acid-inducible gene I
RT	Reverse transcription
RUNX1	Runt-related transcription factor 1
SADS-CoV	Swine acute diarrhea syndrome coronavirus
SARSr-CoV	Severe Acute Respiratory Syndrome-related coronavirus
SeACoV	Swine enteric alphacoronavirus
S	Spike
STAT1	Signal transducer and activator of transcription 1
TBK1	TANK binding kinase 1
TRS	Transcription regulatory sequence
Ty µL	Tylonycteris Microliter
WAG	Whelan and Goldman
